# Telehealth and COVID-19: Empowering Standards of Management for Patients Affected by Phenylketonuria and Hyperphenylalaninemia

**DOI:** 10.3390/healthcare9111407

**Published:** 2021-10-20

**Authors:** Valentina Rovelli, Juri Zuvadelli, Sabrina Paci, Vittoria Ercoli, Alice Re Dionigi, Raed Selmi, Elisabetta Salvatici, Graziella Cefalo, Giuseppe Banderali

**Affiliations:** Clinical Department of Pediatrics, ASST Santi Paolo e Carlo, San Paolo Hospital, University of Milan, 20142 Milan, Italy; juri.zuvadelli@asst-santipaolocarlo.it (J.Z.); sabrina.paci@asst-santipaolocarlo.it (S.P.); vittoriaercoli@gmail.com (V.E.); alice.redionigi@yahoo.it (A.R.D.); raed.selmi@asst-santipaolocarlo.it (R.S.); elisabetta.salvatici@asst-santipaolocarlo.it (E.S.); graziella.cefalo@asst-santipaolocarlo.it (G.C.); giuseppe.banderali@unimi.it (G.B.)

**Keywords:** phenylketonuria, PKU, hyperphenylalaninemia, HPA, telehealth, COVID-19

## Abstract

Phenylketonuria (PKU) and Hyperphenylalaninemia (HPA) are inborn errors of metabolism (IEM) due to mutations in the *PAH* gene resulting in increased blood phenylalanine (Phe) concentrations. Depending on the Phe levels, a lifelong dietary intervention may be needed. During the COVID-19 pandemic, finding new strategies to ensure follow-up and metabolic control for such patients became mandatory and telehealth was identified as the most eligible tool to provide care and assistance beyond barriers. The aim of this study was to evaluate how telehealth use may have impacted disease follow-ups. Seven hundred and fifty-five patients affected by PKU/HPA in follow-ups at the Clinical Department of Pediatrics (San Paolo Hospital, ASST Santi Paolo e Carlo, University of Milan, Italy) were included in this study. The data regarding the used telehealth model, type of performed consultations and patients’ perspectives were retrospectively collected and analyzed after a one-year experience of implemented follow-ups. The results demonstrated that telehealth seemed to be a useful tool to improve the adherence to treatment and that it could guarantee continuous assistance and care beyond the surrounding epidemiological status. Patients expressed great satisfaction with the offered services and requested that they were implemented in standards of care on a long-term basis. Our results suggested the implementation of telehealth in the management guidelines for PKU/HPA patients.

## 1. Introduction

The COVID-19 pandemic strongly impacted the lives of patients affected by chronic diseases, including those affected by inborn errors of metabolism such as phenylketonuria (PKU) and hyperphenylalaninemia (HPA) [1,2,3].

Phenylketonuria and hyperphenylalaninemia (PKU and HPA; OMIM 261600) are inborn errors of metabolism (IEMs) due to mutations in the *PAH* gene, normally coding for the liver enzyme phenylalanine hydroxylase (PAH, EC 1.14.16.1), which converts the aminoacid phenylalanine (Phe) into tyrosine (Tyr) [4]. The absence of or decrease in PAH activity results in increased blood Phe concentrations, or its metabolites, with the possible consequence of toxic levels mainly reaching the CNS. Left untreated, related symptoms can develop shortly after birth and include neurological impairment with possible psychomotor delay, seizures, autism and behavioral disorders. For patients affected by PKU, the current mainstay treatment is a lifelong dietary intervention (made of low-protein foods, amino acid substitutes and micronutrient supplements) able to guarantee normal growth and neurodevelopment. The palatability and ease of use of the products, along with the willingness to adhere to follow-up checks and monitor indications, are primarily important to ensure metabolic control [5]. For patients affected by hyperphenylalaninemia, no dietary/pharmacological intervention is required, still follow-ups may be needed for monitoring purposes, such as for preventing the possible consequences during the pregnancies of affected females [6].

Management guidelines are currently available for healthcare providers (HCPs) dealing with these patients, including recommended monitoring frequencies and the type of necessary assessments. Nevertheless, the COVID-19 pandemic urged the need to change/implement these paradigms as most of assessments could not be performed in the same way due to ongoing ministerial restrictions. In this context, realizing new strategies to ensure follow-ups and metabolic management became mandatory and telehealth was identified as the most eligible tool that could be used to continue to provide care and assistance, despite the pandemic [7,8,9,10,11,12,13,14,15].

The aim of this study was to evaluate the possible outcomes of using telehealth during the pandemic and also if, based on the results, it should be considered as a tool to be implemented in the current management guidelines. The telehealth model used, numbers of visits performed (both “in-person” or as “video consults”) and the satisfaction percentages among patients were retrospectively analyzed and compared among users and non-users after a one-year experience of implemented follow-up. The results suggested that this tool needed to be considered for implementation in the current standards of assistance and care.

Additionally, as PKU/HPA were one of the most diffuse inborn errors of metabolism [16] with hundreds of patients affected worldwide in need of chronic management consisting of frequent assessments [17], our study could add insights and be of interest for other IEM-affected populations for potential future applications.

## 2. Materials and Methods

Data on patients affected by HPA or PKU (with confirmed molecular diagnosis), either on dietary or pharmacological interventions (e.g., Kuvan ^®^), in follow-ups at our Metabolic Clinic (San Paolo Hospital, ASST Santi Paolo e Carlo, University of Milan, Italy), were collected retrospectively (1 April 2019 to 30 March 2021), and analyzed and graphed using Microsoft Excel^®^ software. Statistical analysis was performed using R version 4.0.3.

Both PKU and HPA patients were included in this study based on the fact that, even if for HPAs there is still no general consensus on the required management guidelines, in our clinic the strategy normally includes follow-ups when required. Additionally, in this category, we noticed that it could result in ameliorated outcomes, with patients receiving constant assurance about their metabolic control and the eventual modifiers that may occur, most of all during child-bearing ages which can be better addressed (both for pregnant women who may be in need of dietary interventions and for men who may want to be aware about reproductive risks).

For each patient, data collection included age, race, gender, type of PKU (e.g., HPA or PKU) and type of ongoing treatment. Modalities of obtaining access to the clinic (“in-person” vs. “video consults”), including frequencies, were also retrospectively collected and analyzed. All clinical managements of patients, both for “in-person” and “video consults”, were performed in accordance with the relevant guidelines and regulations.

Patients not returning to the clinic for at least 2 consecutive years at time of study initiation were identified as “lost at follow-up”, as suggested by the literature [18].

The study was conducted in accordance with D.L. 196/2003 (“Italian data protection code”) and the guidelines for the processing of personal data of our clinical institution.

Study period was set accordingly to type of data that would be retrospectively analyzed to avoid any possible bias due to different contexts of occurrence (availability of telehealth vs. non availability), and thus to accurately analyze data related to telehealth use.

Analyzed timeframe for video consulting was set from April 2020–March 2021 (lockdown measures began, in Italy, at the end of February 2020 and telehealth was implemented only afterwards with ongoing measures of containment, including restricted/limited/regulated/numbered access to the hospital). With regard to “in-person” visits, analyzed timeframe was instead set from April 2019 to March 2021, in order to include any possible relevant modifier (e.g., 2019 could be identified as a “normal activity” period, 2020 the “pandemic spread” period and 2021 the “back to normal” period).

Over the study period where video consulting was available, all patients were asked if willing to take advantage of the implemented telehealth service. Two subgroups of patients were consequently identified based on their acceptance to use/nonuse telehealth: “GROUP TU” (“telehealth users”), identified as performing at least 1 video consult (VC) during study period; “GROUP NTU” (“non-telehealth-users”), identified as never using VC but performing at least 1 “in-person” consultation during study period.

The differences between studied groups were determined by Mann–Whitney U or Kruskal–Wallis test. The relation between the two categorical variables was assessed by chi-square test. A *p*-value of <0.05 was deemed as statistically significant.

Categorical variables were expressed as number of frequencies and percentages. Quantitative variables, if normally distributed (Shapiro–Wilks test), were described by mean ± standard deviation; otherwise as median and min–max.

### 2.1. Implemented Telehealth Model within Our Clinic

At our clinic, the current follow-up program for PKU/HPA patients consisted of outpatient visits as needed and annual checks in a day hospital regimen; home monitoring requiring frequency (DBS cards) was different depending on age, metabolic control and type of disease (HPA/PKU) [17]. Due to the limits dictated by the pandemic, such type of clinical management could not be assured. Different models of assistance were thus needed and changes to the habitual setting of care were amended as follows.

**Figure 1:**
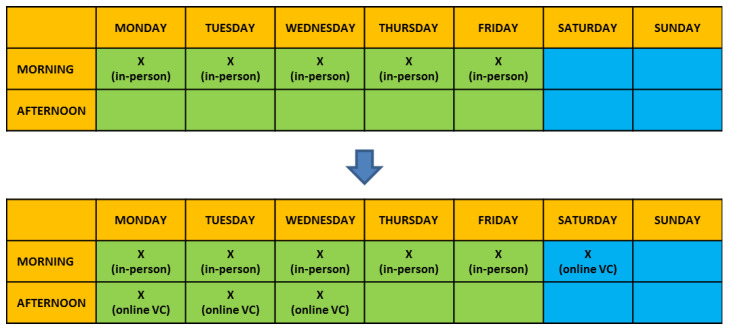
Modified timetable of scheduled appointments. “In-person” appointments were provided of 3 supplemental afternoons available slots for video consulting during the working week and 1 morning over the weekend, for adults patients only.

“In-person” visits, normally distributed over 5 days a week and including only morning time frames with ≈1 h allocated per each consult, were implemented with supplemental available slots for video consulting. This included 3 afternoons per week (during working days) and 1 morning per weekend (meaning Saturday morning, available for adult patients only) [Figure 1]. Time allocated per each video consult (VC) was comparable to “in-person” visits (≈1 h allocated). The healthcare providers (HCPs) team was also implemented with 1 supplemental dietitian specifically committed to the care and needs of adult patients.

Upon the explanation of the implemented service (educational session based on textual documentation sent out by email), video consulting was performed online using Skype ^®^ platform following local regulations (EU Regulation 679/2016 (GDPR) and D.L. 196/2003 as amended by D.L. 101/2018).

**Figure 2:**
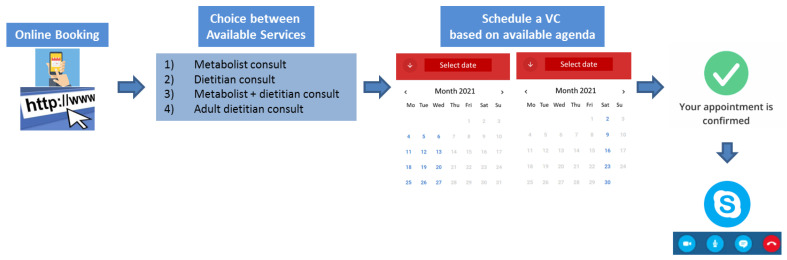
Online flow used for video consulting online assessments and dedicated agenda, including Skype ^®^ platform use upon acquisition of signed informed consent and the different types of possible consult available.

In order to obtain access to the service, patients were asked to sign an informed consent (for underage patients, informed consent was submitted and signed by the parent/legal guardian), which allowed them to self-book appointments over a dedicated online agenda. The latter provided different types of possible video consults to be required based on personal needs [Figure 2] including: (1) “Metabolist consult”: consultation with only the metabolic physician; (2) “Metabolist + dietitian consult”: consultation with both metabolist physician and the dietitian; (3) “dietitian consult”: consultation with the dietitian only; and (4) “adult dietitian”: consultation with a dietitian specialized in dealing with adult patients with PKU. All types of consultations included an interview regarding the state of health and related factors (e.g., school/work outcomes, social/psychological issues, infectious diseases, sport activities, etc.), as well as providing feedback on blood tests performed at home and lab results, where applicable. For dietitian consultations, the interview also included discussions on ongoing dietary regimen and related issues.

When video consulting could not be performed, e.g., adult patients not able to use internet services, absence of sufficient bandwidth for the transmission of the expected data (video/audio), and inadequate tools available at home (no smartphones/PC), the possibility to benefit from a “long-phone-call with Clinic” was also provided to patients, thus making it available to access telehealth services even if only by phone.

When performing VCs, anthropometric measures (weight and height if applicable) were recorded at home by adult patients after appropriate training by the research team. For pediatric patients, parents/legal guardians were asked to note their weight and height at home (after same training by the research team) or to have them recorded by their general practitioner (GP) along with a physical examination, if applicable.

Patients who could not perform “video consults” nor “long-phone-call” and needed access to the clinic were invited to the clinic where the required examinations were performed throughout all the retrospectively analyzed period, aside from the lockdowns and related restrictions.

Home monitoring (DBS sampling) was carried out regularly during all timeframes analyzed, as requested by guidelines [19]. Furthermore, as during lockdown blood sampling (venipuncture) could not always be taken, as they were not identified as essential/of emergency, since, partly assured by DBS monitoring, patients were also offered the possibility of taking blood analysis (other than plasma amino acids) in laboratories close to home, where necessary. With regard to the limitations of dietary adherence often related to the dislike of protein substitutes, during lockdown we also activated a “delivery service system” for protein substitutes, directly to the patients’ pharmacies, with the support of the companies that produced medical foods.

### 2.2. Satisfaction Questionnaire

Type of used consultation, satisfaction percentages, as well as possible other outcomes, were investigated upon submission of a self-made questionnaire coherently realized for our patients via “Google Forms survey^®^” online service. All patients included in this study were asked to take part in the questionnaire, upon acquisition of signed informed consent via email, which asked if they used telehealth services.

The questionnaire consisted of 20 questions, including 17 closed-ended questions (sometimes requiring the numerical expression of the degree of agreement/disagreement, from 0 = highly disagree to 10 = highly agree) and 3 open-ended questions. Questions were designed to investigate satisfaction percentages, advantages and limitations of video consultancy and patients’ perceptions. Filling in the questionnaire was anonymous and required ≈ 20 min for completion. For questions where an opinion had to be given, multiple choices and open answers were also allowed.

Collected data were analyzed and graphed using Microsoft Excel^®^ software. Thematic analysis allowed each answer to be grouped and for results to be expressed as percentages.

## 3. Results

### 3.1. Demographics

A total number of 755 patients, including PKU and HPA, in the follow-up at our clinic were screened and reviewed for inclusion in this retrospective study (*n* = 755, sample size). Among these, 324 were identified as PKU (either on dietary treatment alone or pharmacological therapy) and 431 as HPA. The mean identified age and gender of the study population were nearly equally distributed, as reported in Table 1.

**Figure 3:**
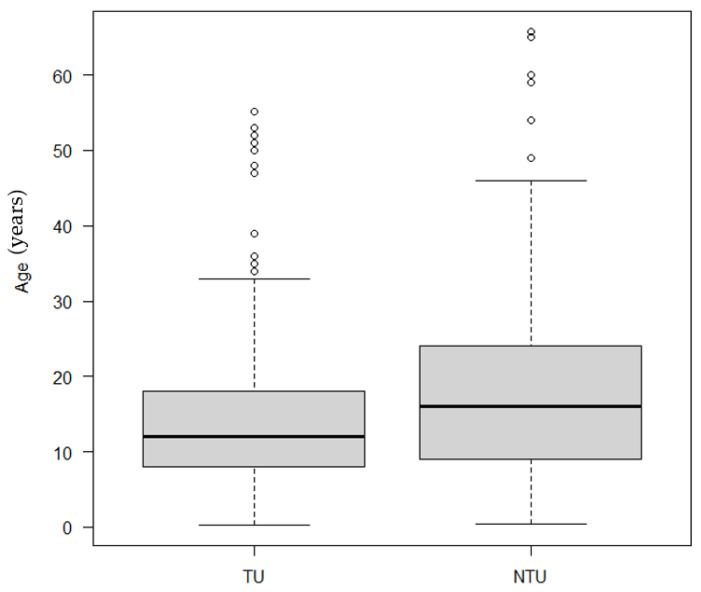
Differences between telehealth users (TU) and non-users (NTU) based on age.

Based on gender, no statistical significance was observed between telehealth users and non-telehealth users (*p*-value = 0.53), even when stratified for PKU/HPA. A statistically significant difference was instead identified between telehealth users and non-users based on age: the average age was significantly lower for telehealth users compared to non-users (*p*-value < 0.0001) (Figure 3).

### 3.2. Video Consults (VC) vs. “In-Person” Visits

Overall, 64% of our population used the telehealth services offered and were identified as “telehealth users” (GROUP TU) as they participated in at least one video consult during the study period; the remaining 36% of patients never used telehealth (GROUP NTU).

A total number of 620 video consults (VCs) were performed over the retrospectively analyzed study period. Among those, 266 were performed for PKUs (with an annual number of accesses per patient varying from 1 to 4) vs. 220 for HPAs (with an annual number of accesses per patient varying from 1 to 2). PKUs, in most cases, took advantage of the offered service (82%), while HPAs demonstrated similar rates of use/nonuse (51% users vs. 49% nonusers).

There were no reported VCs during 2019 and the first months of 2020 (January–March), as VC was not still available to patients; numbers began to rise as we started to implement those services and added them to the clinical practice, with a mean of accesses per month progressively increasing and stabilizing to 51.6 ± 2.1 per month.

**Figure 4:**
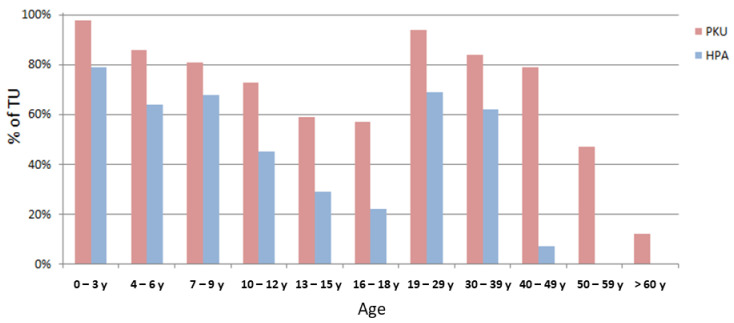
Percentages of telehealth use among PKUs and HPAs based on age (the “age” field indicates the age of the patient even in the case of underage patients, where the parent is the real interlocutor as required by legal regulations).

The percentage of telehealth use varied not only according to the type of disease, but also according to age (Figure 4). While the age ranges of maximum access overlapped among PKU and HPA, being in both cases early ages (0–3 years) and adult ages (20–30 years) with overall higher frequencies for PKUs compared to HPAs, the mean number of accesses differed during the adult ages: HPAs tended to show a marked decrease after the age of 40 years (decreasing even to 0 access/year) while PKUs maintained telehealth use beyond this age. From analyzing such results, we could observe that, within this population, there were 15 “lost-at-follow-up” adult PKU patients who returned to the clinic during the pandemic when asked if willing to perform an online video consult. Additionally, when comparing the current data with the numbers of patients returning to the clinic in previous years (the last 5 years before the pandemic), a higher number of patients could be observed (15 per year vs. a previous mean of 2.7 patients/year).

With regard to “in-person” visits, the rate of patients who did not show up for scheduled visits was lower for VCs than for “in-person” visits (2.4% vs. 8.6%).

The number of visits performed during the “normal activity” period (2019) was 96.2 ± 26.9 per month, covering the required monitoring frequencies as per the management guidelines [9]. Afterwards (2020), briefly during/after the first imposed lockdown in Italy, identified as the “pandemic spread” period, they consistently decreased to 32 ± 29.3 (median 20), reflecting the application of the amended restriction rules. They are now slowly returning to normal and remain substantial stable 95.3 ± 11.4 (September 2020–March 2021).

**Figure 5:**
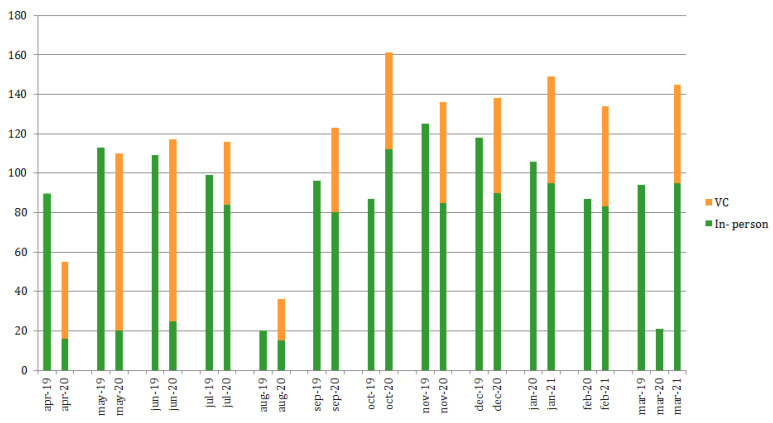
Numbers of visits performed per month (including both “in-person” and video consults, VC) over all retrospectively analyzed time periods (April 2019–March 2021).

The comparison between the number of online vs. “in person” visits performed per month is shown in Figure 5, based on the analyzed timeframe.

### 3.3. Satisfaction Questionnaire: Expectations Meets Reality

There were 451 patients (60% of sample size) who completed the online proposed questionnaire, with 73% TU (*n* = 329) performing at least one video consult during the study period.

In general, telehealth was highly appreciated, with 70% of patients grading it 10/10 and only minor percentages expressing lower rates; however, these rates were never lower than 6/10 (9/10 = 26% and 6/10 = 4%). The aspects that resulted in such positive gradings for the use of telehealth services are expressed in Table 2.

Ninety-eight percent of patients agreed that video consulting seemed to be a useful and reproducible service that they would like to be implemented in the long-term to guarantee and increase the adherence to follow-ups, regardless of the surrounding epidemiological situation. Ninety-six percent of patients also declared that, even if telehealth could not replace “in-person” evaluations, they would like such a tool to be integrated in their habitual follow-up, and thus added to the current standards of recommended monitoring.

With regard to the expression of disapproval, the most reported reasons were:

- “*I prefer the human relationship face to face*”.
- “*As we are online, a clinical visit cannot be performed*”.
- “*I don’t need more visits then the ones already previewed, my type of PKU only need 1 visit per year*”.
- “*Time allocated for video consult was not sufficient to allow me to express all the doubts I had*”.

Among patients identified as NTU, the given reasons for not having used the telehealth service, or even for not having the intention of using it in the future, were mainly related to two standpoints: the timing of slots allocated for VCs (e.g., not sufficiently adaptable to work needs) and available technological tools (e.g., not having smartphones, PC, or a good internet connection).

Sixty-four percent (*n* = 289) of patients who completed the questionnaire were affected by PKU. All the types of available video consults were used, with 40% of patients using the “dietitian visit”. Among these, 65% used the “adult dietitian”, 33% used the “Metabolist visit” and 27% used the “Metabolist + dietitian visit”.

## 4. Discussion

The COVID-19 pandemic, besides the devastating impact it has had all over the world, imposed a new set of challenges from a medical standpoint. We must find new strategies to assure the management and care, not only of patients affected by COVID-19, but also for all those patients affected by other diseases, mainly chronic diseases, who need ongoing medical care, especially patients affected by IEMs. Among these, PKU and HPA populations represent a valuable example since they are numerically significant as a whole and require long-term follow-ups, as described in the current literature [20].

During these difficult times, the need to use new tools to ensure the ongoing treatment and assistance for such patients is urgent, and thus telehealth has become one of the most exploited services, as it directly addresses the gap in healthcare underutilization [11,13,15,20,21,22].

In this paper, the data regarding the used telehealth model, visits performed (both “in-person” or as “video consults”) and patient’s perspectives were retrospectively analyzed and compared among users and non-uses after a one-year experience of implemented follow-ups, to investigate how such new tools were used, who benefits most from them and if they helped HCPs to manage patients overcoming imposed barriers. Consequently, the aim of this study was to evaluate if, based on the results, telehealth should be considered as a tool to be implemented in the current management guidelines.

Telehealth services were widely used among our patients (64% TU), mostly for PKUs. Younger patients demonstrated a greater access to the services, as expected because of greater uncertainties in the management of the disease due to the still recent diagnosis, the possible difficulties in maintaining optimal metabolic control, and the likely fear of growth-related problems. The higher rates of access were again highlighted later on (aged 20–30 years), both for PKUs and HPAs. This may relate to the need for more support for pateints of child-bearing ages, who normally fear and dread the potential metabolic fetal consequences of uncontrolled Phe levels. Interestingly, HPAs and PKUs diverge when patients are in their late 40s. Such results were expected for HPAs, as they obviously lose the need of a continuous follow-up at such stages, but were relatively uncommon with regard to PKUs as we normally expect them to demonstrate lower rates of clinic access while showing an increase in “lost-at-follow-up” situations [18,23,24,25,26]. On the contrary, we could demonstrate that during the pandemic there were more adult patients identified as “lost-at-follow-up” who returned to the clinic when asked if they were willing to profit from the newly implemented services (telehealth) (15 patients were “lost-at-follow-up” vs. a mean of only 2.7 patients/year over the previous 5 years analyzed). De facto, we speculate that adult PKUs may tend to prefer a virtual access to the clinic as it can be best integrated into their work/social lives without the need to take days off work. Additionally, providing an additional dedicated adult dietitian, also available on Saturdays, might also have played a major role in this aspect. Our results suggest that telehealth is more effective in reaching patients who struggle to adhere to the clinic’s face-to-face appointments, and that it may be more suitable for the needs of working adults.

With regard to the number of visits performed, related to the stage of the pandemic, our data show that telehealth was used during the entire pandemic, with its aims changing with the stages of the pandemic. During the early days of the pandemic, where “in-person” visits could not be performed due to ministerial restrictions (excluding emergency situations such as positive newborn screening, pregnancy, etc., for which access to the clinic was always guaranteed, even during lockdowns), it served as a potential substitute, allowing access to health services online and assuring continuous medical assistance beyond distance barriers. Additionally, from a medical standpoint, telehealth services could account for missed in-person visits, boosting them and allowing HCPs to retrieve the estimated previewed visiting numbers. Later on, “video consulting” started to merge with “in-person” visits, leading to the identification of VCs as an additional service, to be used based on both the physician’s and patient’s needs, often unexpressed previously or, in any case, without the possibility of being satisfied from both points of view. This is still true today as telehealth use is still requested and assured to patients and still accounts for many consultations that could previously not be performed any other way. Such results clearly demonstrate the added value of telehealth as it can guarantee the continuous assistance and care beyond the eventual surrounding epidemiological situations; nevertheless, it should not link to the perception that telehealth can replace “in-person” visits, as our ongoing rates of access can confirm that they still constitute an essential part of clinical monitoring (96.16 ± 26.91 accesses per month over 2019 vs. 95.27 ± 11.45 over September 2020–March 2021). Instead, telehealth should be used with different aims based on patients’ needs.

Furthermore, we highlighted that the rates of being absent for scheduled visits were lower for VCs when compared to “in-person” visits over comparable timeframes (2.4% vs. 8.6 %), proving that telehealth may facilitate patients in adhering to the provided indications for their management, despite lockdowns.

All these data together suggest that telehealth implementation should be considered in the current standards of assistance and care in order to improve adherence and monitoring.

Willing to consider other possible related outcomes, in this paper we also wanted to investigate patients’ perspectives in the use of the proposed services. The data from a self-made questionnaire were thus collected aiming to record patients’ thoughts.

Telehealth was highly appreciated among patients, with a grading of 10/10 in 70% of cases (the lowest grade received was 6/10). The satisfaction percentages were justified by different topics such as reducing waiting times and the need to take days off work, and being able to work with the metabolic team in a more protected environment. With regard to disapproval comments, reasons of timing (limited time allocated for VCs) and not being able to perform high-quality assessments (with limits of the available technological supports) were identified as the main factors responsible.

With respect to the type of consultation used, the service from which patients seem to benefit most was the “dietitian visit” (40% of use). This result was in line with the fact that the management of PKU was mainly nutritional, thus, even when not able to provide in-person assessments with clinical evaluations, the management of patients could be implemented, even when consultations were conducted remotely. This result supported the evidence already described in the literature; that a higher staffing intensity was linked to a higher adherence to treatment rates and better metabolic control [1,26].

Ninety-six percent of patients also declared that, even if they did not consider telehealth as a possible replacement for “in-person” evaluations, they would like it to be integrated into their habitual follow-up, agreeing in 98% of cases that it could be useful to provide support, and thus should be implemented in the current standards of care on a long-term basis.

Psychological issues and potential QoL/neurological changes were not assessed in this study as they were not considered the primary aims of the evaluation; further studies may be useful to better evaluate these aspects. Other potential limitations to this study could be that it did not evaluate the possible correlations between the introduction of telehealth and metabolic control, even if there were already published results highlighting the possible positive outcomes [19]. Additionally, the patients answering our questionnaire included both those first taking advantage of video consultancy when other possible alternatives were not available, and those who instead used telehealth for a specific purpose. This could lead to potentially different perspectives when addressing the questions, consequently changing the outcomes. Further studies may be needed to investigate such aspects.

## 5. Conclusions

The world scenario in which we have recently found ourselves operating as healthcare providers has strongly impacted the available standards of clinical practice, especially regarding patients suffering from chronic diseases still in need of follow-ups and treatment during the pandemic. From a HCPs perspective, we strongly believe that the empowerment of interventions that can promote access to care and assistance services, such as telehealth, can play a major role in addressing HCPs and may be strongly beneficial to improve clinical outcomes.

The resulting data from this study demonstrate how much the social context which types of patients are normally exposed to can have a strong influence on the ability to maintain a focus on their diseases, as well as the extent to which this directly links to the intensity of the clinical staffing that can be provided.

Our results highlight how telehealth use can result in positive changes of outcomes for patients affected by chronic diseases, such as PKU and HPA, and add insights to be considered for other IEM-affected populations for potential future applications [1,3,22,26,27]. Telehealth services allow patients to be reached more effectively and quickly, facilitating them to obtain access to the clinic and receive assistance regardless of their geographical location and/or possibilities to travel/take days off work.

As such outcomes can be reproduced in an ongoing scenario, policy decisions should encourage the further utilization of these methods (including ways to realize telemedical consultations within the legal basis of the European Union) to improve healthcare outcomes. Therefore, we suggest that telehealth is added to the current standards of clinical management and guidelines to improve the outcomes of PKU/HPA patients.

## Figures and Tables

**Table 1:** Demographic characteristics of PKU/HPA population screened and reviewed for study purposes.

	Demographics		
	PKU (*n* = 324, 43%)	HPA (*n* = 431, 57%)	Total (*n* = 755)
Age (y) Median (min–max)	18 (0.2–65.8)	12 (0.4–55.1)	13 (0.2–65.8)
Gender (*n*) M/F	186/138	203/228	389/366

**Table 2:** Given the reasons for expressing approval in the use of telehealth services among patients affected by PKU/HPA.

Reasons for Expressing Approval in the Use of Telehealth Services	
*“Lower infectious risk”*	80%
*“I don’t need to move from home”*	76%
*“Seeing my consultants more often makes me feel more “monitored””*	61%
*“I don’t lose a day of school/work”*	40%
*“I can choose the day and time I prefer according to my needs”*	39%
*“I can book a close appointment at any time”*	25%
*“With the video mode I can show my baby in his home context without the limit of the hospital enviroment”*	23%
*“I dont’have to wait my turn in the queue”*	17%
*“Being at home, I feel more comfortable”*	6%

## Data Availability

Data available on request due to privacy restrictions. The data presented in this study are available on request from the corresponding author. The data are not publicly available due to internal regulations.
